# Long Non-Coding RNAs: New Players in Plants

**DOI:** 10.3390/ijms23169301

**Published:** 2022-08-18

**Authors:** Zhennan Zhao, Shoujian Zang, Wenhui Zou, Yong-Bao Pan, Wei Yao, Cuihuai You, Youxiong Que

**Affiliations:** 1 Key Laboratory of Sugarcane Biology and Genetic Breeding, Ministry of Agriculture and Rural Affairs, Fujian Agriculture and Forestry University, Fuzhou 350002, China; 2 Sugarcane Research Unit, USDA-ARS, Houma, LA 70360, USA; 3 Guangxi Key Laboratory for Sugarcane Biology & State Key Laboratory for Conservation and Utilization of Agro Bioresources, Guangxi University, Nanning 530005, China; 4 College of Life Sciences, Fujian Agriculture and Forestry University, Fuzhou 350002, China; 5 Key Laboratory of Ministry of Education for Genetics, Breeding and Multiple Utilization of Crops, College of Agriculture, Fujian Agriculture and Forestry University, Fuzhou 350002, China

**Keywords:** long non-coding RNA, plant growth and development, biotic and abiotic stress, new players

## Abstract

During the process of growth and development, plants are prone to various biotic and abiotic stresses. They have evolved a variety of strategies to resist the adverse effects of these stresses. lncRNAs (long non-coding RNAs) are a type of less conserved RNA molecules of more than 200 nt (nucleotides) in length. lncRNAs do not code for any protein, but interact with DNA, RNA, and protein to affect transcriptional, posttranscriptional, and epigenetic modulation events. As a new regulatory element, lncRNAs play a critical role in coping with environmental pressure during plant growth and development. This article presents a comprehensive review on the types of plant lncRNAs, the role and mechanism of lncRNAs at different molecular levels, the coordination between lncRNA and miRNA (microRNA) in plant immune responses, the latest research progress of lncRNAs in plant growth and development, and their response to biotic and abiotic stresses. We conclude with a discussion on future direction for the elaboration of the function and mechanism of lncRNAs.

## 1. Introduction

In addition to being attacked by various pathogens (e.g., bacteria, fungi, and viruses), plants are also prone to lots of environmental stresses (e.g., drought, high temperature, salt, and low temperature). Plants have evolved several molecular mechanisms that enable them to adapt to these stresses. In eukaryotes, more than 90% of RNA transcripts are termed ncRNAs [1,2] and do not encode proteins. lncRNAs are a key player in regulating various aspects of genomic activities [3]. So far, with the progress of sequencing technology, the Plant Long non-coding RNA Database version 2.0 (PLncDB V2.0) has been constructed with 1,246,372 lncRNAs from more than 80 plant species [4]. Another lncRNA database, NONCODEV6, contains 94,697 lncRNAs from 23 plant species [5]. At the same time, the database of experimentally confirmed functional lncRNAs (EVLncRNAs2.0) contains only 506 lncRNAs [6] (Table 1).

Plants have a variety of transcription machineries. Four DNA-dependent RNA polymerases are believed to be involved in the production of lncRNAs. Unlike mRNA, lncRNAs do not have the potential of protein coding. Regarding gene expression, lncRNAs often function as structural, catalytic, or regulatory molecules [7]. They can affect all elements of a gene, including the promoter, untranslated regions, exons, introns, and the termination region, and thus control the gene expression at different levels, including access, transcription, splicing, and translation [8,9,10,11,12]. Some lncRNAs are involved in protecting the integrity of the genome, while others are engaged in responses to adverse environmental conditions such as temperature fluctuations, drought, and pathogen attacks [13,14,15]. Plants respond to the surrounding environment (sunlight, temperature, water availability, carbon dioxide concentration, etc.) or pathogen attack (fungus, bacteria, virus, etc.) by multiple processes, in which lncRNAs may play key roles [16,17,18].

## 2. Production, Characteristics, Nomenclature, and Classification of lncRNAs

**Figure 1:**
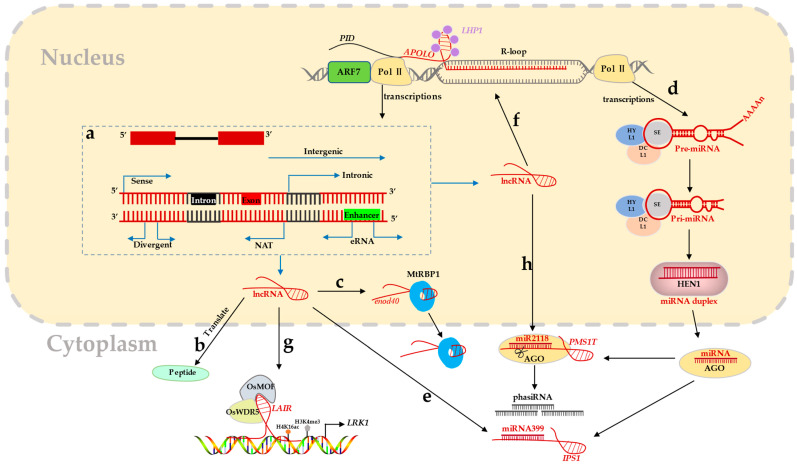
The source and mechanism of lncRNA. (**a**) The lncRNAs are transcribed inside the nucleus from the genome by Pol II and the arrows represent different types of lncRNAs. (**b**) lncRNA can encode small peptides [27]. (**c**) lncRNA *enod40* directly binds to MtRBP1 (*Medicago truncatula* RNA binding protein 1) in root nodules to relocate MtRBP1 from the nuclear speckle of plant cells to the cytoplasmic granules [48]. (**d**) miRNA is transcribed by RNA Pol II. First, the pre-miRNA (precursor miRNA) is processed into miRNA duplex by DCL1 (Dicer-like protein 1), and then the miRNA duplex is processed into single stranded miRNA by HEN1 (HUA ENHANCER 1). The mature miRNA strand is combined with AGO (Argonaute) to carry out post-transcriptional gene regulation through target cutting or inhibition [49]. (**e**) lncRNA *IPS1* (*INDUCED BY PHOSPHATE STARVATION1*) can competitively bind to miRNA399 to upregulate the expression level of *PHO2* and maintain phosphate homeostasis in *Arabidopsis* [50]. (**f**) Transcription of lncRNA *APOLO* (*AUXIN-REGULATED PROMOTER LOOP*) and *PID* is directly activated by ARF7, while *APOLO* binds to its adjacent site *PID* to form an R-loop and recruits *LHP1* to change chromatin conformation. *APOLO* can regulate auxin-related response genes to coordinate auxin distribution and lateral root formation [51]. (**g**) *LAIR* (*LRK Antisense Intergenic RNA*) is an inverted NAT of *LRK* (leucine-rich repeat receptor kinase), which can directly interact with the *LRK1* genomic region and act as a scaffold to recruit OsMOF or OsWDR5 to deposit H4K16ac or H3K4me3, respectively, resulting in up-regulation of *LRK1* expression and increased grain yields [52]. (**h**) lncRNAs *PMS1T* (*PHOTOPERIOD-SENSITIVE GENIC MALE STERILITY T*) and miR2118 combine with *Pms1* (*photoperiod-sensitive genic male sterility 1*) transcript *PMS1T*, which can be recognized by miR2118 and cut to form a string of 21-nt miRNAs. These plant-specific miRNAs are called phasiRNA (phased siRNA), which regulates the fertility of rice [53].

According to the protein coding ability, RNA can be divided into two types, protein-coding and non-protein-coding [7]. Generally, an RNA that encodes protein is called coding RNA (also mRNA), while an RNA that does not encode any protein is called ncRNA [14]. Other than rRNAs and tRNAs, ncRNA can be further divided into sncRNA (small non-coding RNA of ≤50 nt in length) and lncRNA of ≥200 nt in length. So far, there is no formal method for naming different lncRNAs. In general, lncRNAs can be classified into five categories based on the direction and starting site of transcription events: (1) long intergenic ncRNA (lincRNA); (2) intron ncRNA (IncRNA); (3) antisense RNA and natural antisense transcript (NAT); and (4) divergent lncRNA; (5) enhancer RNA (eRNA) [19,20,21] (Figure 1a).

Most non-coding RNAs often lack high sequence or secondary structure conservation, and their higher-order structures are unclear [22]. The biogenesis process of many lncRNAs has a similar pattern to mRNAs, and most lncRNAs are enriched in the nucleus. [23,24]. lncRNA is different from mRNA in many aspects [25]. lncRNAs vary widely in length and contain fewer exons. Similar to mRNAs, lncRNAs usually have an m^7^G cap at the 5′ end and a poly-A tail at the 3′ end. mRNAs are produced by RNA polymerase II (Pol II), while different lncRNAs are generated by different RNA polymerases: Pol II, Pol III, Pol IV, or Pol V. During plant growth and development, the expression and coding capacity of lncRNAs differ from those of mRNAs. lncRNAs are expressed at lower levels than mRNAs [26]. Interestingly, some lncRNAs may contain ORFs (open reading frames) that may have the potential to encode oligopeptides [27] (Figure 1b).

lncRNA, once known as “transcriptional noise”, has been found to play a vital role in various life processes [28,29]. The first study of lncRNA in animals was reported in 1991, in which Brown et al. [30] discovered that lncRNA *XIST* expression could silence the whole X chromosome during development. *MALAT1* (*metastasis-associated lung adenocarcinoma transcript 1*) was identified as a highly expressed ncRNA in lung cancer. The expression level of *MALAT1* was associated with increased metastatic potential and poor prognosis in patients with non-small cell lung cancer [31]. In the animal kingdom, the functional mechanisms of lncRNAs are intensively studied, especially in animal cells [32,33], neural differentiation [34,35], cancers [36], organ development [8], and other fields.

The study of plant lncRNAs is a new field. With the development of sequencing technology, tens of thousands of lncRNAs have been identified. These lncRNAs participate in the regulation of different growth and development processes of plants, such as responses to pests and diseases [37,38,39,40,41], growth [42,43,44], and abiotic stresses [45,46,47].

## 3. The Action Mode and Function Mechanism of lncRNAs

**Figure 2:**
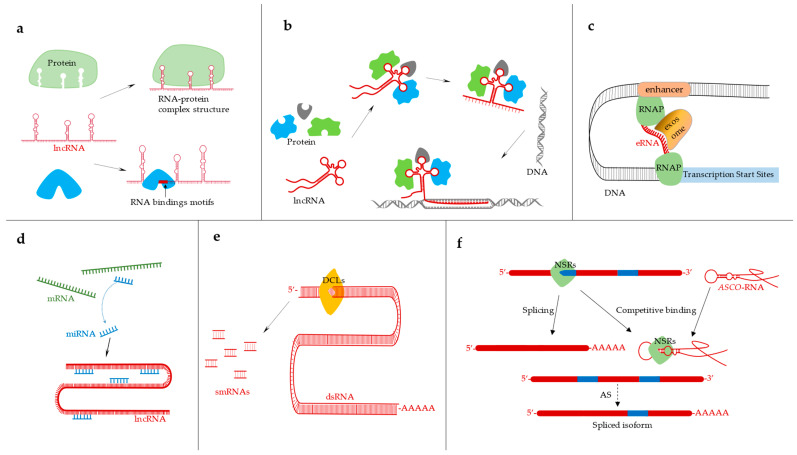
The action mode and function mechanism of lncRNA. (**a**) The expression of lncRNA may require special secondary structure or specific binding motifs. (**b**) lncRNA can act as a molecular scaffold to shorten the distance between different protein complexes and combine with specific sequences to play a special function. (**c**) The eRNAs expressed by enhancers are regulated by exosomes, which can combine with promoters and enhancers to affect the topology of DNA and finally change the expression of genes [61]. (**d**) lncRNA acts as a molecular sponge by adsorbing miRNA to regulate the expression of downstream genes. (**e**) Double stranded lncRNA can be used as a precursor of smRNA (small miRNA). (**f**) *ASCO*-lncRNA (AS competitor long noncoding RNA) in *Arabidopsis* can affect the expression of proteins regulating alternative splicing. *ASCO* acts as a bait to compete with mRNA to bind to NSR (nuclear speckle RNA) splicing regulators. *ASCO*-RNA and NSR-binding proteins compete for the binding of their targets, and hijacking NSR changes for the splicing pattern of mRNA targets regulated by NSR and produces alternative splicing isomers [73].

lncRNAs have been shown to perform many biological functions with complex and varied mechanisms in many eukaryotes [54]. Figure 2 depicts the action mode and function mechanism of lncRNA. They regulate gene expression at the transcriptional and post-transcriptional levels and are involved in epigenetic regulation [7,55,56]. At present, there is no unified statement on the action mechanism of lncRNA. In this review, we systematically summarized the regulatory mechanisms of lncRNAs at different molecular levels, and the regulatory role of lncRNAs in plant stress and growth and development.

### 3.1. Multilayered Regulation of Gene Expression

lncRNA can bind to transcription factors as signal molecules to participate in various regulatory reactions or take part in signaling pathways to further regulate the spatiotemporal expression of protein-coding genes [57]. For instance, the lncRNA *as*-*DOG1* can inhibit the expression of *DOG1* to break the dormancy of *Arabidopsis* seeds [58].

lncRNA can bind to protein [48]. Many lncRNAs are in chromatin and can interact with proteins to promote or inhibit their binding activity in the target DNA region [25]. lncRNA can guide RNA–protein complexes to bind to specific locations or recruit chromatin-modifying enzymes to target genes either in *cis* or *trans* (Figure 2a). For example, in *M. truncatula,* the lncRNA *enod40* could bind to MtRBP1 protein directly in root nodules to relocate MtRBP1 from nuclear speckles to cytoplasmic granules in plant cells [48] (Figure 1c).

lncRNAs can perform molecular functions as scaffold molecules [57,59] (Figure 2b) They can combine with various proteins to form ribonucleoprotein complexes. The specific sites contained in lncRNAs can be combined with certain regulatory molecules, thereby affecting the life process of an organism [60] (Figure 2a). Some enhancer RNAs can even affect DNA topology [61] (Figure 2c). A lncRNA produced by RNA Pol IV in *Arabidopsis* is the binding scaffold for several RNA-binding proteins [62]. According to previous studies, Pol IV is believed to produce siRNA precursors [28]. Pol V can generate scaffold transcripts essential for the recognition of target genes and ultimately chromatin modification by the RdDM (RNA directed DNA methylation) pathway [28]. Unlike Pol IV, Pol V is mostly not required for siRNA biogenesis [28]. However, a subset of siRNAs has been shown to require Pol V, suggesting that it may have a limited or indirect involvement in siRNA biogenesis [28]. The RdDM pathway mainly depends on two core proteins, DCL3 (DICER-LIKE3) and AGO4 (ARGONAUTE4). DCL3 cleaves long double-stranded RNAs to generate siRNAs (small interfering RNAs), which bind to AGO proteins to form AGO–siRNAs complexes, and lncRNAs generated by RNA polymerase act as scaffolds to transport AGO–siRNAs complexes to target chromatin sites [63,64] (Figure 1d).

### 3.2. Interaction between ncRNA and miRNA

lncRNA can be used as a bait to combine with miRNA and then act as a molecular sponge by blocking the interaction between miRNA and its downstream target genes and indirectly regulating the target gene function of miRNA [65] (Figure 2d). Several lncRNAs have been found to be precursors of miRNAs and siRNAs [66,67] (Figure 2e). miRNA is ncRNA with a length of 20–24 nt. miRNAs are Dicer nuclease processed derivatives of immediate precursor pre-miRNAs, they contain a hairpin structure and have a 5′-phosphate and a 2-nucleotide 3′ overhang [68], and the further mature miRNA single-strands bind to AGO through targeting dot cleavage or repressing post-transcriptional gene regulation [49,68]. On one side, miRNA targets lncRNA to generate phasiRNA (phased small interfering RNA) [69]. On the other side, lncRNA acts as sources of miRNA or regulates miRNA accumulation or activity at the transcriptional and post-transcriptional levels [70]. The most important action mode of lncRNA and miRNA is to reduce the expression level of miRNA by adsorbing miRNA to reduce the inhibition of mRNA and dynamically regulating the translation speed and stability of downstream target genes [71]. For example, both *IPS1* (Figure 1e) and *At4* can competitively bind to miR399 to upregulate the expression level of PHO2. miR399 and PHO2 play an important role in maintaining phosphate homeostasis in *Arabidopsis* [50,72]. Such fine-tuning of miRNA activity by endogenous non-cleavable lncRNA targets is referred to as targeting [50].

## 4. lncRNAs Are Involved in Regulating Plant Growth and Development

### 4.1. Plant Vernalization

**Figure 3:**
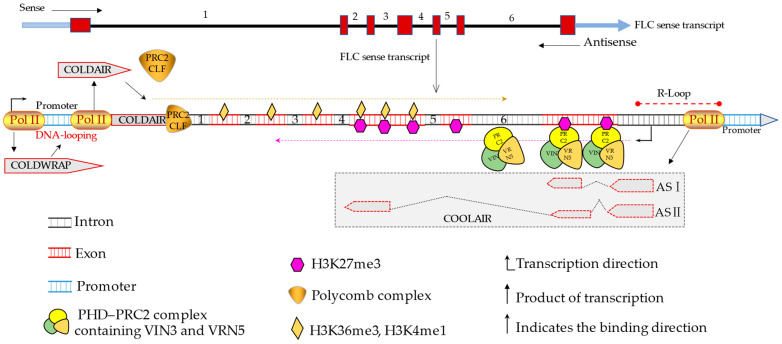
*COOLAIR*, *COLDWARP*, and *COLDAIR* are involved in regulating vernalization response of *FLC* gene [19]. *COLDAIR* is transcribed from the first intron of *FLC*. It inhibits the establishment of H3K27me3 and induces H3K4me3 by recruiting and directing PRC2-CLF to *FLC* [78]. *COLDWRAP* is from the promoter of *FLC* induced by vernalization. *COLDWRAP* and *COLDAIR* collaborate to establish a restrictive intracellular chromatin loop [79]. *COLDWRAP* binds to PRC2-CLF, localizes to *FLC* gene, and promotes vernalization of *FLC* chromatin in response to H3K27me3 [79]. *COOLAIR* has two alternatively spliced isoforms (AS I and AS II). R-loop stabilization mediated by AtNDX inhibits *COOLAIR* transcription, thereby altering *FLC* expression [83]. The nucleation region consists of VIN3, VRN5, and PRC2, and accumulates as part of the PHD–PRC2 complex downstream of the FLC transcription start site [81]. The aggregation of the nucleation region leads to a decrease in H3K4me3/H3K36me3 and an increase in H3K27me3 [82]. AS: alternative splicing; PRC2: polycomb repressive complex 2; green boxes indicate exons of *COOLAIR*, AS I and II represent exons, dotted lines indicate splice sites; the red dotted line represents the R-loop; the yellow and purple dotted lines indicate the modification direction; numbers represent introns; H3K4me1, histone H3 lysine 4 monomethylation; H3K36me3, histone H3 lysine 36 trimethylation; H3K27me3, trimethylation of histone H3 lysine 27.

lncRNA is involved in the vernalization of plants [59,74]. Figure 3 illustrates how *COOLAIR*, *COLDWARP*, and *COLDAIR* are involved in regulating vernalization response of *FLC* gene. In *Arabidopsis*, *FLC* (*FLOWING LOCUS C*) encodes a mad box transcription factor, a key gene regulating vernalization [75]. *FLC* transcription will be inhibited at a low temperature but gradually decreased with the extension of cold exposure [76]. lncRNA *COOLAIR* (*COLD-INDUCED LONG ANTISENSE INTRAGENIC RNA*) is the antisense transcript of *FLC*, which is involved in the methylation of H3K36 and the synchronous replacement of H3K27m3 in the early vernalization [77]. *COLDAIR* (*COLD-ASSISTED INTRONIC NONCODING RNA*) is transcribed from the first intron of *FLC* and directed to *FLC* by recruiting the polycomb complex PRC2–CLF to inhibit the establishment of H3K27me3, while H3K4me3 is induced at the *FLC* locus to promote the enhancement of *FLC* expression [78]. *COLDWRAP* (*WINTER-INDUCED NONCODING RNA FROM THE PROMOTER*) mainly controls the intragenic gene loop between the promoter and the first intron of the *FLC* gene [79]. When exposed to a cold stress, *COLDWRAP* and *COLDAIR* work together to establish a restrictive intracellular chromatin loop that inhibits *FLC* expression [79]. In addition, *COLDWRAP* combines to PRC2-CLF to help it locate in the *FLC* gene and promote H3K27me3 response vernalization of *FLC* chromatin [79]. When induced by cold treatment, *COOLAIR* can cover almost the whole *FLC* gene [80]. During cold exposure, the nucleation region composing of VIN3, VRN5, and PRC2 accumulates as part of the PHD–PRC2 complex downstream of the FLC transcription initiation site [81]. In this region, the aggregation of this complex will lead to the decrease in H3K4me3/H3K36me3 and the increase in H3K27me3 [82]. *COOLAIR* appears in the form of multiple alternative splicing isomers and indirectly inhibits *FLC* expression through transcriptional interference [80]. A recent report has found a homologous domain protein, AtNDX, which regulates the expression of *COOLAIR* [83]. AtNDX binds to single stranded DNA rather than double stranded DNA non-sequentially in vitro and is in the heterochromatin region of the *COOLAIR* promoter in vivo [83]. The R-loop mediated by AtNDX stably inhibits *COOLAIR* transcription, thereby changing *FLC* expression [83]. This region extends from 200 bp upstream of the *COOLAIR* promoter to the polyadenylation site near *COOLAIR* [83]. In conclusion, these lncRNAs jointly participate in and regulate the vernalization response of *Arabidopsis*.

*VRN1* is a flowering activator and a central gene regulating the vernalization of cereal crops [84]. Winter wheat flowering requires long-term low-temperature induction, and *VRN1* is a key regulator of low-temperature induction and can accelerate the flowering transition [74]. lncRNA *VAS* from the wheat *VRN1* gene can recruit transcription complexes RF2b–RF2a to enable it to bind to the TaVRN1 promoter region to activate VRN1 transcription and promote flowering [74].

### 4.2. Plant Growth

lncRNAs take part in plant growth and regulate plant life activities in seed development [84,85], fiber accumulation [13], lipid metabolism [86], and leaf development [87]. In rice, lncRNA *TL* (*TWISTED LEAF*) was reported to maintain leaf flatness by regulating the expression of the *R2R3-M*YB gene [88]. In alfalfa, the lncRNA *enod40* binds to MtRBP1 in root nodules to relocate the protein from the nucleus to play a role in the cytoplasm [52]. Nitrate is a key signal molecule that regulates plant gene expression, metabolism, growth, and development [89,90,91,92]. The lncRNA *T5120* was reported in *Arabidopsis* and can promote nitrate assimilation and plant growth, thereby improving nitrogen utilization efficiency [93]. The overexpression of *T5120* in *Arabidopsis* promoted the plant response to nitrate with enhanced nitrate assimilation, improved biomass, and root development. It is noteworthy that *T5120* is co-regulated by the nitrate transcription factor NLP7 and the nitrate sensor NRT1.1 to regulate nitrate signal transduction [93]. miR9678 targets the lncRNA *WSGAR* in wheat and produces phasiRNA by cutting, which delays seed germination [69]. *ASCO*-lncRNA in *Arabidopsis* plays a role as a bait and regulates root development [73]. In *Arabidopsis*, *ASCO* expression affects the splicing patterns of several mRNA targets and is regulated by NSRs binding proteins. Therefore, *ASCO*-lncRNA can hijack nuclei as regulators to produce alternative splicing isomers, causing changes in plant root development [73] (Figure 2f). lncRNA *APOLO* can coordinate auxin distribution and lateral root formation [51] (Figure 1f).

### 4.3. Light Response

**Figure 4:**
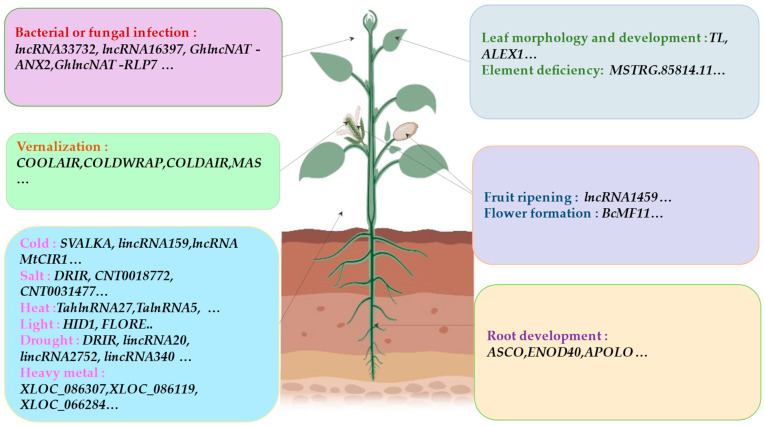
The role of lncRNA in plant growth and stress response. *lncRNA33732* and *lncRNA16397* respond to pathogen infection [97,98]. *GhlncNAT-ANX2* and *GhlncNAT-RLP7* are a pair of lncNRAs that regulate pathogenic infection and are involved in enhancing cotton disease resistance [99]. *COOLAIR* [77], *COLDWRAP* [79], *COLDAIR* [59], and *MAS* [100] respond to cold and regulate spring flowering time in *Arabidopsis*. *SVALKA* regulates cold signal transduction [101]. The binding of *lincRNA159* to miR164 reduces the expression of three *NAC* genes targeting miR164 in cassava under cold stress [102]. *HID1* plays an important role in seedling photomorphogenesis under red light [94]. *FLORE* regulates photoperiod flowering [95]. *DRIR* regulates plant tolerance to drought and salt stress [103]. *CNT0018772* and *CNT0031477* respond to salt stress [104]. *TalnRNA5* and *TahlnRNA27* respond to heat stress [105]. *XLOC_086307, XLOC_086119*, and *XLOlC_066284* are involved in heavy metal cadmium response [106]. *MSTRG.85814.11* regulates iron deficiency response [107]. *lncRNA1459* is involved in fruit ripening [108]. *ASCO* alters root development [73]. *APOLO* coordinates auxin distribution and lateral root formation [51]. *enod40* promotes root nodule formation [48].

Among the few lncRNAs with known biological functions, two are involved in the light regulation process. *HID1* (*HIDDEN TREASURE 1*) is involved in photomorphogenesis and seedling greening [94]. *FLORE* (*CDF5 LONG NON-CODING RNA*) is a lncRNA that regulates circadian rhythm. The aggregation of *FLORE* can inhibit the expression of *CDF5* (*CYCLING DOF FACTOR*), while CDF can directly bind and repress the CO (CONSTANS) and FT (FLOWERING LOCUS T) promoters to regulate photoperiod flowering [95]. It is interesting that both CDF5 and *FLORE* transcripts accumulate in vascular tissues to conversely regulate the CO-FT module, which in turn regulates the flowering time [95]. Strong light can enhance the synthesis and coloration of anthocyanins in apple fruits. Qiu et al. [96] verified that a lncRNA *MdLNC610*, which is located 81 kb downstream of the ethylene biosynthesis gene *MdACO1*, was involved in anthocyanin accumulation under strong light. *MdLNC610* can promote ethylene release and anthocyanin accumulation in apples upstream of *MdACO1* [96]. Both strong light and ethylene can significantly promote apple coloring and anthocyanin biosynthesis [96]. *MdLNC610* can enhance the activity of the *MdACO1* promoter and is in the same topological domain of *MdACO1*. *MdLNC610* and *MdACO1* can significantly improve ethylene release, anthocyanin accumulation, and the expression of related genes [96]. Figure 4 enumerates the roles of lncRNAs in plant growth and stress responses.

### 4.4. Yield and Seed Formation

lncRNAs affect seed formation and yield composition. lncRNA *LAIR*, a reverse antisense transcript of LRK1, was identified in rice [52]. It can directly interact with the *LRK1* genomic region and act as a scaffold to recruit OsMOF and OsWDR5. H4k16ac and H3K4me3 were deposited, resulting in the up regulation of *LRK1* expression and the increase in grain yield [52] (Figure 1g). Chen et al. [109] have found the lncRNA *MISSEN* that regulates the molecular functions of tubulins during endosperm nuclear division and endosperm cellularization. By competing with tubulin, *MISSEN* binds to HeFP and prevents HeFP (helicase family protein) from participating in endosperm development, which in turn interferes with the normal development of the endosperm, rendering the produced seeds defective.

### 4.5. Floral Organ Development

At present, many lncRNAs, including *LDMAR* (*LONG-DAY SPECIFIC MALE-FERTILITY-ASSOCIATED RNA*) [110] and *PMS1T* (Figure 1h) [53], are known to be involved in the regulation of flower growth and development. In *Arabidopsis,* the upregulation of *LINC-AP2* and the downregulation of its neighboring gene *AP2* (*APETALA2*), an intergenic lincRNA close to the transcription factor *AP2*, occur simultaneously after *TCV* (*turnip crinkle virus*) infection [9]. The strong upregulation of *LINC-AP2* is correlated with structural abnormalities of flowers [9]. Another lncRNA, *XLOC_057324*, plays an essential role in controlling fertility and flowering [111]. The lncRNA *SUF* (*SUPPRESSOR OF FEMINIZATION*), an antisense lncRNA of *MpFGMYB*, is important for Goldilocks female sexual differentiation. *SUF* loss of function mutants generated by the deletion of Cas9 null mutants shows male to female sexual conversion [112]. The identification of *ncRNAW6* in the *HaWRKY6* promoter revealed another regulation layer of this gene by ncRNAs [113]. *ncRNAw6* is derived from a transposon of the mite family that is capable of forming a hairpin structure. The hairpin is processed by DCL3 to produce 24-nt het siRNAs to trigger the DNA methylation of the *HaWRKY6* region and enhance *HaWRKY6* transcription [113]. The level of DNA methylation, loop formation, and the level of *HaWRKY6* expression are regulated in a tissue-specific manner [113]. *Ef-cd*, an antisense RNA at the *OsSOC1* locus, positively regulates ossoc1 activity through depositing H3K36me3 and reducing the time span required for plant maturation, but not reducing the yield [114]. An intronic lncRNA *AG-incRNA4* in *Arabidopsis* is expressed in leaves and interacts with the PRC2 complex component *CLF* to deposit the H3K27me3 histone mark at the *AG* loci, thereby contributing to the repression of *AG* expression in leaves [115]. The knockdown of *AGlincRNA4* leads to the activation of *AG* in leaves by reducing the H3K27me3 levels at *AG* sites. The corresponding mutants exhibit a phenotype such as ectopic *AG* expression [115]. During cabbage pollen development and pollination fertilization, 15 lncRNAs were predicted to potentially regulate the expression of 13 miRNAs in the form of ETMs (endogenous pseudo target mimics). Two of these lncRNAs, *bra-eTM160-1* and *bra-eTM160-2*, were further identified to regulate the activity of cabbage miRNA160, which is involved in pollen development by affecting the expression of ARF family members of target genes [116]. These studies have demonstrated that lncRNAs regulate reproductive growth versus flower bud differentiation at different molecular levels, which is essential for normal plant reproduction.

## 5. LncRNAs Respond to Biotic and Abiotic Stresses

### 5.1. Biotic Stress Response

**Figure 5:**
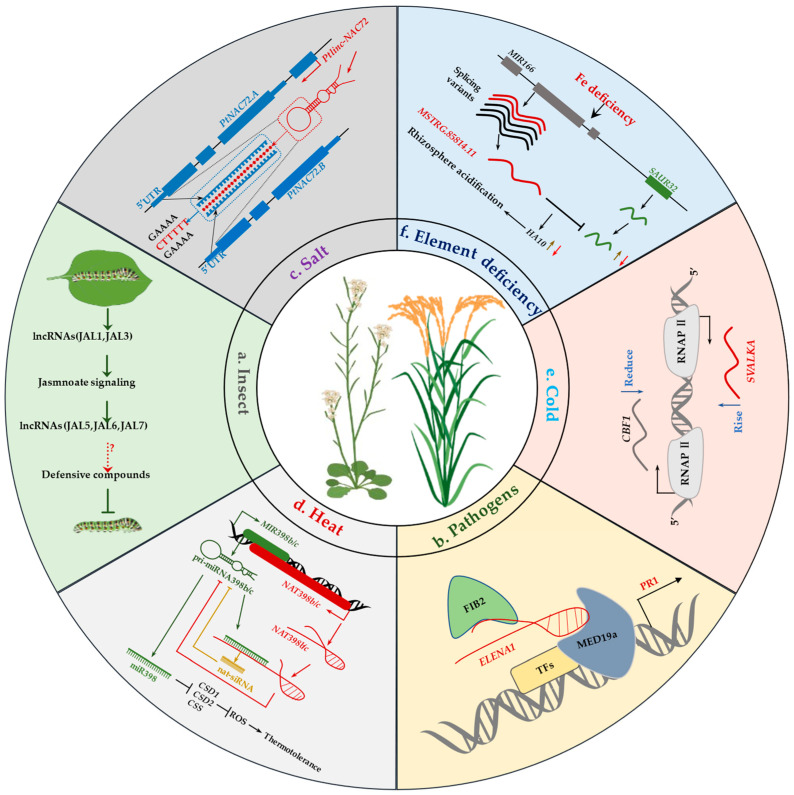
Mechanisms of lncRNAs in response to external pressure. (**a**) JA signaling is regulated by lncRNAs (such as JAL1 and JAL3) in response to early plant attack by diamondback moth [38]. (**b**) The transcription level of lncRNA *ELENA1* increases under pathogen attack. The transcripts then bind to *FIB2* and MED19a (Mediator subunit 19a). When FIB2 dissociates, MED19a then binds to the promoter region to activate the expression of PR1 leading to enhanced disease resistance [117,118]. (**c**) The lncRNA *Ptlinc-NAC72* is induced under long-term salt stress to regulate salt tolerance with the tandem in the *PtNAC72.A*/*B* 5′ UTR [60]. (**d**) *MIR398b*/*c* and its antisense *NAT398b*/*c* genes are co-expressed in vascular tissues. *NAT398b*/*c* inhibits pri-miRNA processing, while knocking out *NAT398b*/*c* promotes miR398 processing. By silencing miR398-targeted genes, heat tolerance is improved. On the contrary, overexpression of miR398 activates *NAT398b*/*c* and reduces heat tolerance. Moreover, *NAT398b*/*c* can also be activated by *MIR398b*/*c* overexpression [69]. (**e**) Prolonged cold exposure peaked in *CBF1* expression along with increased expression of the lncRNA *SVALKA* in the antisense direction to *CBF1*. The transcripts of *SVALKA* would lead to decreased *CBF1* transcription and increased RNA PII occupancy on both strands. *CBF1* repression by RNA PII collisions originates from the *SVALKA*-*asCBF1* lncRNA cascade, ultimately resulting in decreased *CBF1* transcription on the sense strand and decreased full-length *CBF1* mRNA, and thus reduces cold tolerance [101]. (**f**) The spliceosome *MSTRG.85814.11* positively regulates its target gene *SAUR32* to promote the response to iron deficiency in the rhizosphere of plants [107].

Plants are attacked by various pathogenic organisms, especially viruses, fungi, and bacteria. Pathogens interfere and destroy the physiological activities of plants in many ways, resulting in a great impact on growth and production. In Figure 5, we show the action mechanism of lncRNA in response to various stresses. To cope with this adverse effect, plants have evolved lncRNA survival strategies [38]. Some lncRNAs are related to the response to herbivorous insect feeding in plants [39]. Some lncRNAs are even associated with insect resistance mediated by the plant jasmonate hormone signal pathway [38]. Some early responding lincRNAs are co-expressed with many genes in the JA signaling pathway [38]. Furthermore, during infestation by phytophagous insects, silencing two lincRNAs (*JAL*1 and *JAL*3) reduces the JA content and the content of insect resistant substances regulated by JA, leading to the weakening of host resistance to phytophagous insects [38]. It is worth noting that the expression of some late responding lincRNAs can also be regulated by the JA signal pathway [38] (Figure 5a).

The lncRNA *MSTRG.19915*, a natural antisense transcript of the MAPK gene *BrMAPK15*, was found to be associated with susceptibility to downy mildew (*Hyaloperonospora brassicae*) in Chinese cabbage [119]. BrMAPK15 enhanced resistance against downy mildew [119]. When *MSTRG.19915* was silenced, seedlings showed enhanced resistance to downy mildew, which may be related to the up-regulation of *BrMAPK15* expression [119]. Li et al. [37] first reported 565 lncRNAs responsive to nematodes, which play a crucial role in host resistance or sensitivity to nematode infection. Zhang et al. [99] extracted the lncRNA *L2* (*GhlncNAT-ANX2*) and lncRNA *L3* (*GhlncNAT-RLP7*) from cotton that were responsive to two major species of *Verticillium dahlia*. Silencing *L2* and *L3* may up-regulate the expression of *LOX1* and *LOX2*, thus enhancing the resistance of cotton to *Verticillium dahlia* [99]. Overexpression of the lncRNA *ELENA1* (*ELF18-INDUCED LONG NONCODING RNA 1*) in *Arabidopsis* increased the expression of *PR1* (pathogenesis-related gene 1) and enhanced the resistance to *Pst* DC3000 (*Pseudomonas syringae pv. tomato* DC3000) [117]. The lncRNA *ELENA1* had increased the transcript level upon pathogen infection and combined with FIB2 and MED19a [117,118]. After dissociation of FIB2, MED19a could continue to bind to the promoter to activate *PR1* expression to enhance disease resistance [117,118] (Figure 5b). *TYLCV* (*tomato yellow leaf virus*) has a great effect on tomato crop production. In *TYLCV*-susceptible strains, *SILNR1* is a key lncRNA for virus resistance and normal leaf development. *SILNR1* is complementary to siRNA produced by *TYLCV*, and *SILNR1* is downregulated to increase host susceptibility [120]. Yu et al. [121] discovered 567 lncRNAs from *Xanthomonas oryzae*-infected rice leaves, the targets of which were significantly enriched with the JA pathway. To reveal the interaction between lncRNAs and JA-related genes, 39 JA-related protein coding genes were found to interact with 73 lncRNAs by co-expression analysis, indicating the potential regulatory role of these lncRNAs in the JA pathway [121]. The lncRNA *ALEX1*, whose expression was highly induced upon pathogen infection, was identified. The overexpression of *ALEX1* in rice caused the activation of the JA pathway and thereby enhanced the host resistance to pathogenic bacteria [121]. As a positive regulator, *lncRNA33732* in tomato was able to enhance tomato resistance against *Phytophthora infestans* by inducing the expression of respiratory burst oxidase and increasing H_2_O_2_ accumulation [97]. In rape, lncRNAs play a significant role in resisting infection of *Sclerotinia sclerotiorum* [122]. Li et al. [123] reported 5294 lncRNAs that were used to construct the expression profiles of lncRNAs responsive to *Fusarium oxysporum* infection in banana. Table 2 lists the lncRNAs research progress and corresponding functional identification in recent years.

### 5.2. Abiotic Stress Response

Many chemical products have entered crop production, which inevitably cause a lot of heavy metal poisoning (such as cadmium, manganese, and lead), and these heavy metals are becoming one of the important hazards in crop production [132]. Under Cd stress, 120 lncRNAs that may regulate genes of *cis* cysteine-rich peptide metabolism, as well as secondary metabolites of trans cysteine rich peptide metabolism and photosynthesis, were identified to activate various physiological and biochemical responses in response to excess Cd, presumably playing important roles in those gene and protein pathways in response to Cd stress [106].

Soil salinization remains a constraint to the increasing global food production. During growth and development, plants suffer from salt stress with reduced yield due to the absorption of too many toxic ions [133]. Wan et al. [134] reported 172 lncRNAs responsive to salt stress through *cis* or *trans* interactions with important coding genes. A total of 35 differentially expressed lncRNAs were predicted to interact with 42 differentially expressed coding genes [134]. These genes may participate in the auxin response and the ABA and Ca^2+^ signal transduction pathways under salt stress [134]. Twelve lncRNAs were predicted to be the target mimics of 17 known mature miRNAs in *Camellia sinensis*, thus affecting the expression of downstream functional genes [134]. A new intergenic lncRNA was identified in *Populus tomentosa*, which was mainly localized in the cytoplasm [60]. *Ptlinc-NAC72* contained a stem ring with five tandem repeats of “CTTTTT” motif, which were complementary to the “GAAAA” repeats in the 5′ UTR of the two target genes [60]. Through recognition and interaction with the salt-responsive element “GAAAA”, *Ptlinc-NAC72* regulated the expression of the two target genes *PtNAC72.A* and *PtNAC72.B* at the same time [60]. Co-transformation and GUS staining have verified that *Ptlinc-NAC72* binds to the 5′ UTR region of two target genes at the post transcriptional level and plays a role in stabilizing gene expression [60]. In addition, stable overexpression of the *Ptlinc-NAC72* gene in *Arabidopsis* can enhance the salt resistance of *Arabidopsis* seedlings [60] (Figure 5c). In cotton, *lncRNA354* is a lncRNA from the intergenic region that acts as an miRNA sponge to participate in the regulation of biological processes [135]. *lncRNA354* affects the response of upland cotton to salt stress by interacting with miR160b. The splicing of the *GhARF17/18* gene maintains normal growth and development. However, under salt stress, *lncRNA354* expression is weakened and the binding of miR160b to *lncRNA354* is decreased, while the increase in miR160b will inhibit the expression of *GhARF17*/*18*, thereby enhancing the resistance to salt stress [135].

Extreme environments cause inevitable hazards to plants. Under these environments, plants generate molecular signals to cope with the stress. In *Arabidopsis*, *MIR398b*/*c* and its antisense *NAT398b*/*c* can interact to regulate plant heat tolerance [70] (Figure 5d). Qin et al. [103] reported a lncRNA *DRIR* (*DROUGHT INDUCED lncRNA*), from *Arabidopsis* that can be induced by ABA, drought, and salt stress. *DRIR* can positively regulate plant tolerance to drought and salt stress by regulating the expression of key genes for stress responses. Also in *Arabidopsis*, lncRNA *SVALKA* can regulate cold tolerance in *Arabidopsis* [101] (Figure 5e). In cassava *(Manihot esculenta Crantz)*, *CRIR1* (a cold-responsive intergenic lncRNA 1) is a positive regulator of the plant response to cold stress [136]. *CRIR1* is significantly induced by cold treatment to interact with MeCSP5 (cassava cold shock protein 5) [136]. Further studies have found that *CRIR1* may recruit MeCSP5 to improve the translation efficiency of mRNA. *CRIR1* affects the mechanism of the cold stress response by regulating the expression of stress response genes and increasing their translation efficiency [136]. In apple, 13 variable spliceosomes for lncRNAs *MSTRG.85814* were identified, of which five were involved in the iron deficiency response. It was further confirmed that the spliceosome *MSTRG.85814.11* could positively regulate its target gene *SAUR32* to promote the plant rhizosphere response to iron deficiency and stepwise regulation by MSTRG 85814.11-SAUR32-H^+^-ATPase (AHA10) in iron deficiency response in an apple graft complex [107] (Figure 5f). StCDF1 (CYCLING DOF FACTOR 1) is a transcription factor that regulates potato (*Solanum tuberosum*) tuberization [136]. StCDF1 and NAT *StFLORE* together regulate water loss by affecting stomatal growth and diurnal opening [137]. Moreover, both natural mutations of *StFLORE* transcripts and CRISPR-Cas9 mutations increase the sensitivity of plants to water restriction [136]. StCDF1 regulates the expression of *StFLORE* and a high level of *StFLORE* expression can reduce water loss and enhance drought tolerance [137].

## 6. Concluding Remarks

lncRNAs, play a role in the process of light morphogenesis, growth and development, stress adaptation, and so on. Although more and more data suggest that lncRNAs also play an important role in plant immunity, the research on its specific regulation mechanism is still limited. The conservation of lncRNAs is not high, and the mechanism revealed in model plants may not be directly applied to other plant species. Therefore, lncRNA research is still in the initial stage of exploration. In the present review, an indispensable role of lncRNAs in plant growth and development, as well as under biotic and abiotic stress, was summarized. A single gene may be regulated by multiple ncRNAs and lncRNAs may not function in a single way or alone. On the contrary, lncRNAs can interact with many genes and proteins and the mechanism is complex. It is worth noting that the structure, function, and origin of lncRNAs in animals and plants are highly similar and there are certain rules to follow [138]. The research of animal lncRNAs can be used as a reference for plant lncRNA. Once target lncRNAs are excavated at a large scale in a specific species, they can be annotated and predicted by using bioinformatic means. Further development of CRISPR/cas9, RNA pull-down, RIP, CHIP, and RNAi may facilitate the elaboration of function and mechanism of the lncRNAs.

## Figures and Tables

**Table 1:** Validated long non-coding RNAs in 10 plant species [6].

Species	Number of Functional lncRNAs
*Arabidopsis thaliana*	160
*Oryza sativa*	43
*Digitalis purpurea*	29
*Zea mays*	26
*Solanum lycoperscium*	24
*Setaria italica*	19
*Populus tomentosa*	18
*Manihot esculenta*	17
*Salvia miltiorrhiza*	17
*Populus trichocarpa*	15

**Table 2:** Discovery and function analysis of lncRNAs.

Time	Name	Species	Biological Functions	References
1997	*TPSI1*	*Solanum lycopersicum*	Phosphate homeostasis	[124]
2002	*GmENOD40*	*Glycine max*	Root nodules formation	[125]
2004	*At4*	*Arabidopsis thaliana*	Phosphate homeostasis	[72]
	*Enod40*	*Medicago truncatula*	Nuclear-cytoplasmic re-localization Root nodules formation	[48]
2007	*IPS1*	*Arabidopsis thaliana*	Phosphate homeostasis	[50]
2010	*MtNOD40*	*Medicago truncatula*	Root nodules formation	[126]
2011	*COLDAIR*	*Arabidopsis thaliana*	Vernalization flowering	[59]
2013	*BcMF11*	*Brassica campestris*	Flowering regulation	[127]
2014	*HID1*	*Arabidopsis thaliana*	Seedling photomorphogenesis	[94]
	*APOLO*		Auxin response; lateral root development	[128]
	*ASL*		Flowering	[129]
2016	*TCONS_00061773*	*Solanum lycopersicum*	Nitrogen-deficient response	[130]
2017	*COLDWRAP*	*Arabidopsis thaliana*	Vernalization flowering	[79]
	*lncRNA16397*	*Solanum lycopersicum*	Disease resistance response	[98]
	*LAIR*	*Oryza sativa*	Rice grain yield	[52]
2018	*TL*	*Oryza sativa*	Leaf shape remodeling	[88]
	*MAS*	*Arabidopsis thaliana*	Vernalization flowering	[100]
	*COOLAIR*	*Arabidopsis thaliana*	Vernalization flowering	[77]
2019	*lncRNA39026*	*Lycopersicon esculentum*	Disease resistance response	[131]
2020	*lncRNA MISSEN*	*Oryza sativa*	Seed development	[109]
2021	*Ptlinc-NAC72*	*Populus trichocarpa*	Salt stress regulation	[60]
2022	*MdLNC610*	*Malus pumila*	Fruit coloring	[96]

## Data Availability

Not applicable.
